# Multicolor photonic patterns through an intensity-controlled single photopolymerization step†

**DOI:** 10.1039/d2cc04050f

**Published:** 2022-08-30

**Authors:** Yari Foelen, Nieké J. M. van Gils, Mart D. T. Claessen, Albertus P. H. J. Schenning

**Affiliations:** Stimuli-responsive Functional Materials and Devices, Department of Chemical Engineering and Chemistry, Eindhoven University of Technology P.O. Box 513 5600 MB Eindhoven The Netherlands a.p.h.j.schenning@tue.nl; Institute for Complex Molecular Systems, Eindhoven University of Technology, Den Dolech 2 5600 MB Eindhoven The Netherlands; SCNU-TUE Joint Laboratory of Device Integrated Responsive Materials (DIRM), South China Normal University, Guangzhou Higher Education Mega Center 510006 Guangzhou China

## Abstract

The UV intensity during photopolymerization allows control over the structural color of a cholesteric liquid crystal (CLC) polymer photonic coating in a single step. Simultaneously, the glass transition temperature (*T*_g_) of the polymer can be tuned by the applied UV intensity. Most likely the low intensity photopolymerization increases the inhibition time, leading to *in situ* formation of polymer fragments through oxygen inhibition. The formation of polymer fragments changes the matrix during the inhibition time, which results in a color change before the polymer network is formed. Additionally, these fragments inside the network act as a plasticizer, effectively lowering the *T*_g_. This method can be combined with temperature responsive properties based on shape memory to fabricate photonic coatings with multiple, responsive colored patterns. The presented work allows for new functionalities in responsive photonic polymers as multiple colors and response temperatures can be incorporated in a single polymerization step.

## Abstract 2

The UV intensity during photopolymerization allows control over the structural color of a cholesteric liquid crystal (CLC) polymer photonic coating in a single step.

## Graphical abstract

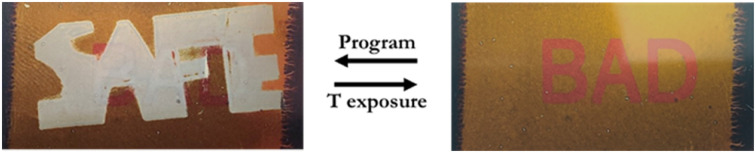

Cholesteric liquid crystal (CLC) polymers are an appealing class of photonic crystals that can be used in applications including responsive coatings for indicators and anti-counterfeiting labels.^1^ Liquid crystal (LC) molecules obtain a CLC phase by adding a small amount of a chiral compound (dopant) to the nematic phase, which induces a helical twist of the rod shaped molecules that inherently possess an anisotropic refractive index.^2,3^ This results in the formation of a helical structure through the depth of the layer, which reflects light with single-handed circular polarization due to the periodic alternation of the refractive index.^4^ Selective wavelengths of the incident light will be reflected, depending on the helical pitch (*P*), the average refractive index (*n*) and the angle of incident light (*θ*), according to Bragg's law as *λ* = *n*.*P*. cos(*θ*).^2,5^ The ability of a chiral dopant to induce a twist is quantified by the helical twisting power (HTP, *β*) which is related to the pitch by *β* = 1/(*c*.*P*) in which *c* is the concentration of the dopant. The HTP is determined by the molecular structure of the dopant and the molecular composition of the LC matrix.^6,7^ CLC polymers can be fabricated *via* free radical photopolymerization of a CLC mixture containing liquid crystal monomers with a reactive end group such as (meth)acrylates.^8^ Such acrylate mixtures are prone to oxygen inhibition during photopolymerization (*vide infra*).^9^

Multicolor patterning of a CLC photonic polymer coating traditionally requires multiple polymerization steps, fixating a specific color in each polymerization step.^10–13^ For example, a two-step procedure applies step growth oligomerization of the liquid crystal molecules, which changes the matrix. The matrix change influences the HTP of the chiral dopant and the reflected color. The resulting colors are fixated in a network during multiple photopolymerization steps.^14^

Multicolor photonic coatings are typically static. However, by making use of the shape memory effect^15,16^ a multicolor temperature-responsive photonic coating can in principle be created. Shape memory photonic polymers can be mechanically deformed into a specific shape when heated beyond their glass transition temperature (*T*_g_) and are fixated in this temporary shape by quenching below the *T*_g_. The original shape is recovered by temperature exposure to their *T*_g_ or above.^17–19^ Previous research on optical time temperature integrators (TTIs) showed a single colored shape memory CLC polymer in which an embossed temporary surface topography created a scattering state that prevented the reflection of red light.^20^ Exposure of the film above the *T*_g_ resulted in a time dependent recovery of the red color.

This work presents a facile method to produce a multicolor CLC polymer with multiple *T*_g_s in a single photopolymerization step. Creation of multicolor photonic materials by a single processing step allows for effortless printing of multicolor patterns or images. We show that by combining these patterns with temperature responsive properties based on shape-memory, photonic coatings can be obtained with responsive multicolored patterns. Our results allow for new functionalities in photonic polymers as multiple colors and response temperatures can be incorporated in a single step.

A CLC mixture (mixture 1), composed of multiple LC molecules, is used to obtain a printable photonic coating with a cholesteric phase at room temperature. Mixture 1 includes chiral dopant 1, difunctional crosslinker 2, monofunctional acrylates 3 and 4, a difunctional thiol 5, and photoinitiator 6 as previously described in the literature (Fig. 1a).^20^ For the exact composition of the mixture, see Table S1 (ESI†). Dithiol 5 acts as a chain extender *via* radical thiol-acrylate polymerization, thereby reducing the crosslinking density and lowering the *T*_g_.^21,22^ The composition of the mixtures was chosen such that a reflective colored coating in the visible spectrum was obtained, but in principle any color is possible by varying the chiral dopant concentration.^23^ The mixture was dissolved in cyclopentanone as a solvent to enable gravure, roll-to-roll printing, and a surfactant (0.1 wt%) was added to promote planar alignment at the coating-air interface.^24^ The mixture was printed on a black flexible biaxially oriented polyethylene terephthalate (PET) substrate, which supports the color contrast. After printing, the solvent was evaporated at elevated temperature (70 °C) for 5 minutes, and afterwards the coating was cooled to room temperature, which induced the CLC phase showing a green color. The printed coatings were then cut into strips of 5 × 2 cm and photopolymerized with UV light in a nitrogen box, resulting in non-sticky and highly flexible polymeric CLC coatings with a bright color. The thickness of the coating was ∼3.5 μm, as measured from a cross-section SEM image (Fig. S1, ESI†). The UV polymerization intensity was controlled by a LED module, by altering the LED distance to the coated substrate, and by placing a Neutral Density (ND) filter with various optical densities between the lamp and coated substrate (Fig. 1b). The UV lamp irradiance spectrum significantly matches the initiator absorbance spectrum (Fig. S2, ESI†).

**Fig. 1:**
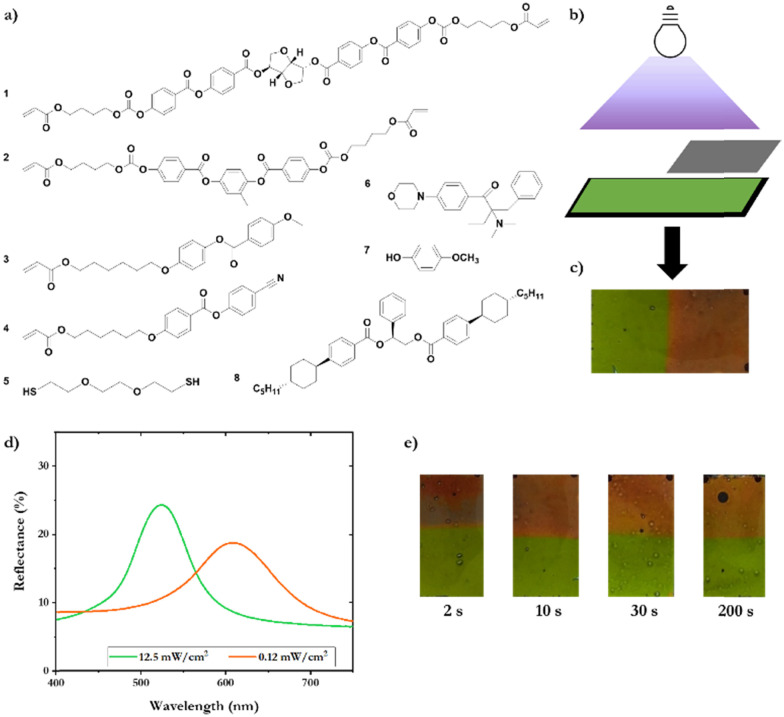
(a) Chemical structures of the molecules used in the CLC mixtures. b) Schematic polymerization process: intensity controlled by an ND filter to expose one part of the coating to 12.5 mW cm^−2^ and the other part to 0.12 mW cm^−2^ in one photopolymerization step. (c) Photograph of the coating, demonstrating the color difference after polymerization for 5 minutes of mixture 1 at two different intensities. (d) UV-Vis reflectance spectra of the polymer coating based on mixture 1, after photopolymerization for 5 minutes at 12.5 mW cm^−2^ and 0.12 mW cm^−2^. (e) Photographs after a range of polymerization times (2–200 s), showing that the colors are formed within 2 s.

To study the role of the light intensity on the photopolymerization reaction, the CLC coatings were exposed to two different intensities for 5 minutes. One part of the coating was exposed to 12.5 mW cm^−2^ UV-A light. An ND filter with ND = 1 (1% transmission) was used to expose the other part of the coating to 0.12 mW cm^−2^ UV-A light (Fig. 1b). After polymerization for 5 minutes, the UV intensity difference results in a coating with a visible color difference (Fig. 1c). In the case of a high intensity, a green color is observed, while in case of a low intensity, a red color is visible. The coating is characterized with UV-vis spectroscopy, and the part photopolymerized with high UV intensity (12.5 mW cm^−2^) demonstrates a reflectance peak around 520 nm (green), while the part that was photopolymerized at low UV intensity using a filter (0.12 mW cm^−2^) displays a reflection peak around 610 nm (red) (Fig. 1d). Furthermore, the polymerization time has no observable influence on the structural colors: the polymerization times were varied from 2 to 200 seconds, demonstrating that the color difference is created within 2 seconds (Fig. 1e). However, it was noted that both polymer patterns were tacky for polymerization times up to 30 seconds, and therefore the coatings are polymerized for 5 minutes to yield maximum conversion. The Fourier transformed infrared spectroscopy (FT-IR) spectrum of both parts of the polymer coating shows the absence of the acrylate C=C peaks (1640, 1410 and 985 cm^−1^), indicating successful photopolymerization after 5 minutes (Fig. S3, ESI†). Differential scanning calorimetry (DSC) of the polymer coating indicates a difference in the glass transition temperature (*T*_g_); the polymer photopolymerized at high UV intensity expresses a broad *T*_g_ around 22 °C while the low intensity photopolymerized polymer expresses a broad *T*_g_ around 11 °C (Fig. S4, ESI†). This reveals that the *T*_g_ of the polymer can be tuned by the applied UV intensity.

The instant appearance of different colors before full conversion can be obtained, indicating that the effect happens during the initiation phase. Literature has shown that the radicals formed during initiation react with monomers to form propagating radical polymer fragments, which undergo oxygen inhibition within the first period (<1 s) of the polymerization until the dissolved oxygen is scavenged.^25^ Further research has illustrated that low intensity decreases the rate of primary radical production during photoinitiation, resulting in a lower polymerization rate and a longer inhibition time.^26^ Simultaneously, the increased inhibition time leads to a slower vitrification of the reaction medium. Likely, in our case the inhibited polymer fragments change the matrix composition and therefore the HTP of the chiral dopant is decreased by the presence of polymer fragments before the polymerization fixates the system.^7^ Therefore, the UV intensity dependent red shift effect is rooted in the initiation phase of the photopolymerization due to inhibition of the dissolved oxygen in the monomer coating and directly correlated to the irradiation intensity. The inclusion of the inhibited polymer fragments might act as a plasticizer inside the polymer network, effectively lowering the *T*_g_ (Fig. S3, ESI†). At high intensity, the rate of polymerization is higher, and the inhibition time decreases, resulting a faster vitrification with no color shift effect.^26^

To validate that the intensity effect is determined by the inhibition time during polymerization, other influences of polymerization kinetics were excluded by using a different CLC mixture (mixture 2) containing chiral dopant 8, mono-acrylate 3 and 4 wt% photo-initiator 6. In the previous mixture 1, chiral dopant 1 acts both as a chiral dopant and as a diacrylate that takes part in the polymerization process. Therefore, in mixture 2, the chiral dopant was replaced by a chiral dopant without polymerizable end groups, chiral dopant 8. Additionally, the mixture was further simplified by excluding diacrylates, to exclude differences in polymerization kinetics of reactive end groups. Also, dithiol (5) was excluded to prevent oligomerization reactions. After polymerization of mixture 2, the intensity dependent red shift is still observed (Fig. 2a). This shows that the red shift is indeed caused by the change in the surrounding matrix and not a result of structural change of the dopant or differences in the polymerization kinetics between reactive end groups. As the intensity dependent red shift is caused in the initiation step, lowering the concentration of the initiator and increasing the inhibition by adding an inhibitor, directly influences the inhibition time and thereby the degree of the red shift. To verify this hypothesis, a new mixture 3 was prepared. Mixture 3 includes molecules 1–6, 4 wt% of radical inhibitor 7 and 0.5 wt% of initiator (instead of 4% in mixture 1) (Fig. 1a and Table S1, ESI†). Indeed, an increase in the red shift created by different polymerization intensity is observed as mixture 1 shows a 90 nm difference in maximum reflection peaks, while mixture 3 shows a difference of 136 nm (Fig. 2b).

**Fig. 2:**
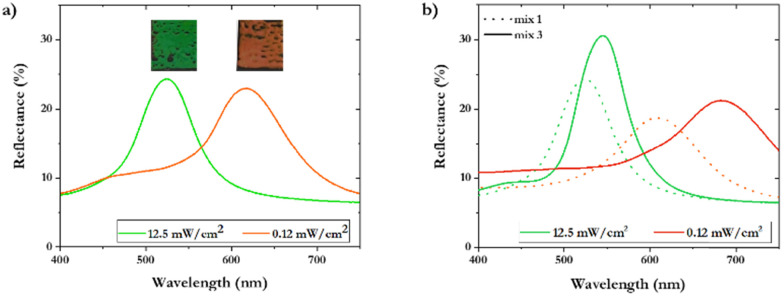
(a) UV-Vis reflectance spectra demonstrating the red shift in a monoacrylate coating (mixture 2) after photopolymerization, with photographs of the coatings. (b) UV-Vis reflectance spectra comparing the extent of red shift after photopolymerization, influenced by the initiator difference between mixture 1 and mixture 3.

Next, we tested if the intensity dependent red shift enables the production of a multicolor pattern in a single photopolymerization process. To produce a coating with colors ranging from blue to red, mixture 3 was used with a higher chiral dopant concentration to produce a blue colored coating at high intensity. An ND step filter with increasing optical density areas (0.2, 0.3, 0.4, 0.5, 0.8, 1) results in a stepwise decrease of the UV intensity. After photopolymerization, a multicolored coating showing blue, green and red colors was produced (Fig. 3a). UV-Vis reflectance measurements demonstrate the shift of the reflection peaks with a very broad reflection band created by the lowest intensity (Fig. 3b). The broadening might be attributed to the absorption gradient created throughout the coating at such low UV intensity. Below an intensity of 0.02 mW cm^−2^, photopolymerization results in a coating that remains a viscous liquid, indicating that no substantial polymerization occurred. Expressing the reflection peaks as a function of the applied UV intensity reveals that the biggest shift takes place in a narrow range at low intensity, where the inhibition time significantly increases (Fig. 3c). As expected, the *T*_g_ of the different polymer regions follows a similar trend: the *T*_g_ decreases down to 4 °C due to the plasticization effect of the increased presence of inhibited polymer fragments inside the polymer created during photopolymerization with low UV intensity (Fig. 3c).

**Fig. 3:**
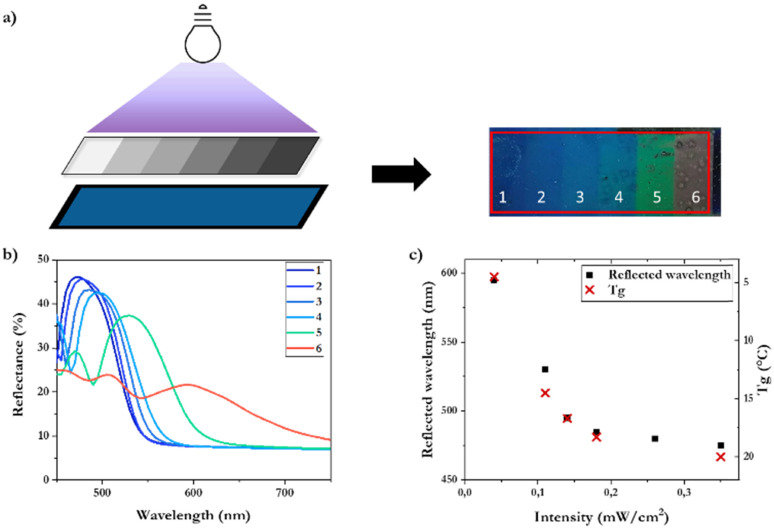
(a) Photopolymerization with an ND step filter to create multiple UV intensities, and the resulting multicolored polymer coating on a flexible black BOPET substrate. (b) UV-Vis reflectance spectra demonstrating the red shift of each differently exposed region. (c) Reflected wavelength peaks (left) and polymer *T*_g_ (right) as a function of the applied UV intensity during photopolymerization.

The presented color patterning method allows for versatile design features of optical indicators by creating multicolor designs. The following demonstrator serves as an example of how patterning can provide additional information when applied in optical TTI labels based on the shape memory effect.^20^

Fabrication of arbitrarily colored patterns in a CLC optical coating can now be done by using a photomask that alters the polymerization intensity. We fabricated a photomask stating the word ‘BAD’ on a transparent polyester film with printing ink, applied by a standard inkjet printer. The difference in transmission between the polyester film and the printed ink created a color difference after photopolymerization of a photonic coating, creating the word ‘BAD’ (Fig. 4). Next, the imprinted pattern was concealed by surface embossing of the CLC coating surface with a stamp in the shape of the word ‘SAFE’. The embossing procedure resulted in concealing the word ‘BAD’ as it prevented the reflectance of a specific wavelength due to a scattering surface.^20^ Exposure to a temperature above *T*_g_ resulted in the disappearance of the embossed temporary ‘SAFE’ pattern as the coating surface restored (Fig. 4 and Movie S1, ESI†). Further research is needed to study the time and temperature dependent restoration of the embossed pattern as it is defined by the surface properties.

**Fig. 4:**
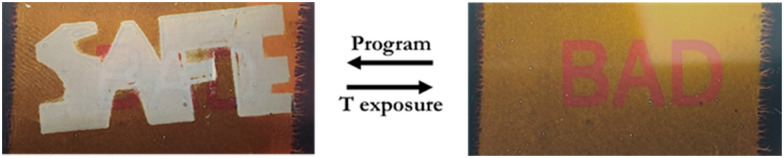
TTI application example. Imprinting of the word ‘BAD’ in a CLC coating (right) which can be concealed by programming the word ‘SAFE’ (left) through surface embossing of a shape memory coating with a stamp. The surface restores during temperature exposure above *T*_g_ and changes the message of the coating from ‘SAFE’ to ‘BAD’.

Intensity-controlled photopolymerization of CLC mixtures enables fast, single step fabrication of multicolor patterns in a polymer film. The observed red shift created during photopolymerization at low UV intensity is most likely caused by the *in situ* formation of inhibited polymer fragments due to dissolved oxygen. At low UV intensity, the inhibition time is considerably longer which increases the concentration of inhibited polymer fragments, affecting the matrix dependent HTP of the CLC mixture before fixation by polymerization. This inhibition effect is applicable to oxygen sensitive radical polymerizations, and therefore independent of composition.^26^ The polymerization intensity controlled red shift results in a lower *T*_g_ due to the plasticization effect caused by the inhibited polymer fragments. This facile method can be combined with temperature responsive properties based on shape memory to fabricate photonic coatings with multiple, responsive colored patterns. This allows for new functionalities in shape memory time-temperature optical indicators as multiple colors and response temperatures can be incorporated in a single step.

The manuscript was written through contributions of all authors. All authors have given approval to the final version of the manuscript.

This research was carried out under project number A17022 in the framework of the Research Program of the Materials innovation institute (M2i) (https://www.m2i.nl) supported by the Dutch government. This research received funding from the Netherlands Organization for Scientific Research (NWO) in the framework of the Innovation Fund Chemistry and from the Dutch Ministry of Economic Affairs and Climate Policy in the framework of the PPP allowance.

## Conflicts of interest

There are no conflicts to declare.

## Supplementary Material

CC-058-D2CC04050F-s001

CC-058-D2CC04050F-s002

## References

[cit1] Foelen Y., Schenning A. P. H. J. (2022). Adv. Sci..

[cit2] Mulder D. J., Schenning A. P. H. J., Bastiaansen C. W. M. (2014). J. Mater. Chem. C.

[cit3] Ohm C., Brehmer M., Zentel R. (2010). Adv. Mater..

[cit4] Coates D. (2015). Liq. Cryst..

[cit5] Kinoshita S., Yoshioka S., Miyazaki J. (2008). Rep. Prog. Phys..

[cit6] Van De Witte P., Neuteboom E. E., Brehmer M., Lub J. (1999). J. Appl. Phys..

[cit7] Cook M. J., Wilson M. R. (2000). J. Chem. Phys..

[cit8] Lub J., Broer D. J., Hikmet R. A., Nierop K. G. (1995). Liq. Cryst..

[cit9] Liu D., Broer D. J. (2014). Langmuir.

[cit10] Zhang P., Kragt A. J. J., Schenning A. P. H. J., De Haan L. T., Zhou G. (2018). J. Mater. Chem. C.

[cit11] Zhang W., Froyen A. A. F., Schenning A. P. H. J., Zhou G., Debije M. G., de Haan L. T. (2021). Adv. Photonics Res..

[cit12] Li Y., Wang M., Urbas A., Li Q. (2013). J. Mater. Chem. C.

[cit13] Xu L., Zhang H., Wei J. (2019). Photochem. Photobiol. Sci..

[cit14] van Heeswijk E. P. A., Yang L., Grossiord N., Schenning A. P. H. J. (2020). Adv. Funct. Mater..

[cit15] Lendlein A., Sauter T. (2013). Macromol. Chem. Phys..

[cit16] Safranski D. L. , Introduction to Shape-Memory Polymers, Elsevier Inc., 2017

[cit17] Espinha A., Serrano M. C., Blanco Á., López C. (2014). Adv. Opt. Mater..

[cit18] Belmonte A., Pilz da Cunha M., Nickmans K., Schenning A. P. H. J. (2020). Adv. Opt. Mater..

[cit19] Yang J., Zhao W., Yang Z., He W., Wang J., Ikeda T. (2019). ACS Appl. Mater. Interfaces.

[cit20] Nickmans K., van der Heijden D. A. C., Schenning A. P. H. J. (2019). Adv. Opt. Mater..

[cit21] Brien A. K. O., Cramer N. B., Bowman C. N. (2006). J. Polym. Sci., Part A: Polym. Chem..

[cit22] Godman N. P., Kowalski B. A., Auguste A. D., Koerner H., White T. J. (2017). ACS Macro Lett..

[cit23] Taugerbeck A., Booth C. J. (2014). Handb. Liq. Cryst..

[cit24] Kulkarni S., Thareja P. (2017). Surf. Rev. Lett..

[cit25] Luu T. T. H., Jia Z., Kanaev A., Museur L. (2020). J. Phys. Chem. B.

[cit26] Christmann J., Ley C., Allonas X., Ibrahim A., Croutxé-Barghorn C. (2019). Polymer.

