# Supplementary material for: Multicolor photonic patterns through an intensity-controlled single photopolymerization step
Source: Chem Commun (Camb). 2022 Aug 30;58(77):10833–6. doi: 10.1039/d2cc04050f (PMC9514011; doi:10.1039/d2cc04050f)
Supplement: CC-058-D2CC04050F-s001 [file CC-058-D2CC04050F-s001.pdf]

Electronic Supplementary Material (ESI) for ChemComm.
This journal is © The Royal Society of Chemistry 2022

Supporting Information

Multicolor photonic patterns through an intensity-controlled single photopolymerization step

Yari Foelen[1], Nieké J. M. van Gils[1], Mart D.T. Claessen[1], Albertus P. H. J. Schenning[1,2,3,]*

**Table S1**	**Mixture composition**
**Figure S1**	**SEM image**
**Figure S2**	**FT-IR spectra of monomer and polymers**
**Figure S3**	**DSC measurements**
**Figure S4**	**UV lamp irradiance and initiator absorption spectra**
**Materials and methods**	**Experimental details**

Table S1. Mixture composition (*compositional change to produce a blue reflecting CLC phase).

Compound	Mixture 1 (wt %)	Mixture 2 (wt %)	Mixture 3 (wt %)
**1**	4	0	4 (5*)
**2**	6	0	3,5
**3**	52	92	56 (55*)
**4**	35	0	31
**5**	3	0	3
**6**	4	4	0,5
**7**	0	0	2
**8**	0	8	0

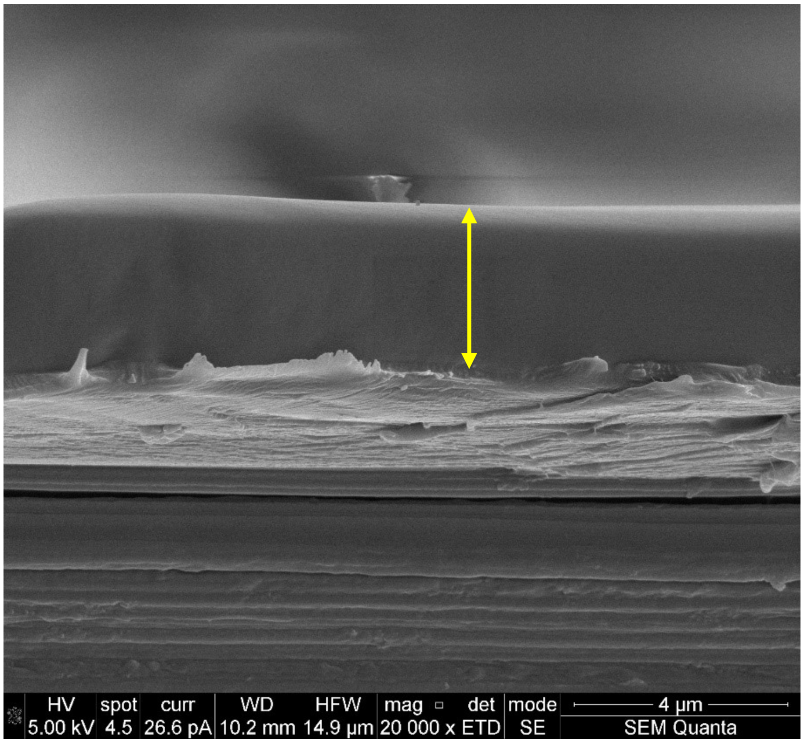

Figure S1. SEM image: a cross section of a polymer coating from mixture **1** on a PET substrate, indicating an average coating thickness ~3.5 µm.

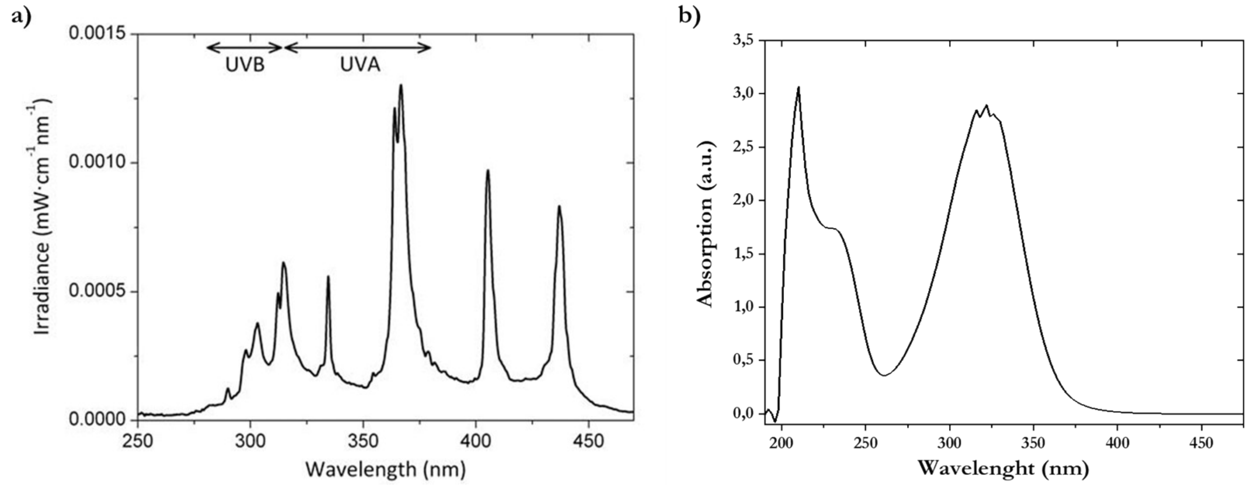

Figure S2. a) Spectrum of the OmniCure 2000 UV lamp irradiance (from specifications) b) Absorption spectrum of Initiator **6**.

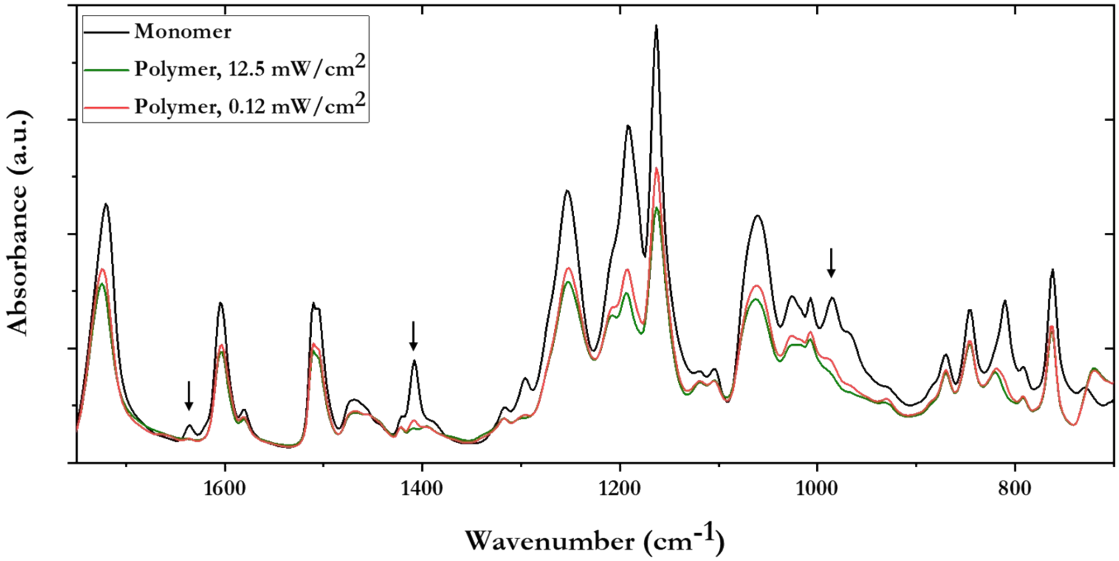

Figure S3. FT-IR spectra of monomer mixture **1** and the parts of the polymer coating polymerized at high (12.5 mW/cm^2) and low (0.12 mW/cm^2) UV intensity.

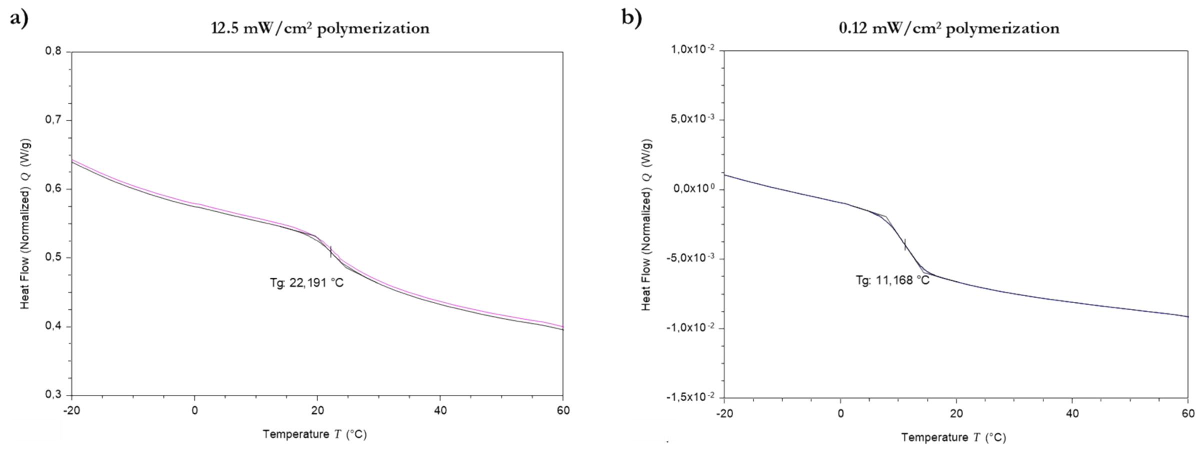

Figure S4. DSC measurements of photopolymerized mixture **1**, a) showing a Tg around 22°C for the (green) coating polymerized at 12.5 mW/cm^2 and b) a Tg around 11°C for the (red) coating polymerized at 0.12 mW/cm^2.

Materials and methods.

Materials: (3R,3aS,6R,6aS)-hexahydrofuro[3,2-b] furan-3,6-diyl bis(4-((4-(((4-(acryloyloxy)butoxy)carbonyl)oxy)benzoyl) oxy)benzoate) [**1**] and 2-methyl-1,4-phenylene bis(4-(((4-(acryloyloxy)butoxy) carbonyl)oxy)benzoate) [**2**] were obtained from BASF. 4-((6-(acryloyloxy)hexyl)oxy)phenyl 4-methoxybenzoate [**3**] was purchased from Synthon Chemicals. 4-cyanophenyl 4-((6-(acryloyloxy) hexyl)oxy)benzoate [**4**] and (S)-1-phenylethane-1,2-diyl bis(4-((1s,4S)-4-pentylcyclohexyl)benzoate) [**8**] were obtained from Merck. 2,2′-(ethane-1,2-diylbis(oxy))bis(ethane-1-thiol) [**5**] and 4-methoxyphenol [**7**] was purchased from Sigma-Aldrich. The photoinitiator 2-benzyl-2-(dimethylamino)-1-(4-morpholinophenyl) butan-1-one [**6**] was obtained from IGM resins. Surfactant BYK-361 N was purchased from BYK. Cyclopentanone was purchased from Acros. Neutral Optical Density filters were purchased from Thorlabs.

Ink preparation: The components were dissolved in cyclopentanone with surfactant BYK-361 N (0.1 wt%) (2:1 solids:solvent ratio), and the resulting solutions were filtered through a 0.2 µm PTFE syringe filter.

Gravure Printing: The ink was printed at a speed of 0.5 m s−1 on black flexible biaxially oriented PET substrates (Tenolan OCN0003, 36 µm thickness) using an IGT F1 printability tester in a gravure printing mode. Afterward, the solvent was allowed to evaporate for ≈2 min at 70 °C and cooled for 60s at room temperature to align the CLC phase.

Polymerization: Photopolymerization was carried out with an Omnicure series 2000 EXFO lamp in a nitrogen box, additional ND filters (Thorlabs) were used to tune the exact UV intensity. The stepwise ND filter was applied with a ND of 0.1, 0.2, 0.3, 0.4,0.5, 0.8, 1.0.

Embossing: A modified hot-stamp machine from KBA-Metronic GmbH was used to compress the surface of the coating into a rubberized metal sheet (Ra ≈ 1 µm) for 30 s at a pressure of 4.5 bar. The machine was fitted with a brass stamp heated to 40°C.

Characterization: UV intensity of the polymerization setup was measured with a radiometer RM12 from Opsytec Dr. Gröbel with a UV-A (400-315 nm) sensor. The reflection of the CLC coatings was measured through ultraviolet−visible spectroscopy, using a PerkinElmer LAMBDA 750 with a 150 mm integrating sphere over a range of 400−750 nm. A Varian 670 FT-IR spectrometer with slide-on ATR (Ge) was used to record IR spectra. Thermogravimetric analysis was performed in a TA Instruments TGA Q500 with a constant heating rate of 5 °C/min. Thermal transitions of the liquid crystalline coatings were analyzed by differential scanning calorimetry using a TA Instruments DSC Q2000 calorimeter with constant heating and cooling rates of 10 °C/min. GPC was performed on a Shimadzu Prominence-I LC-2030C high-performance liquid

chromatography (HPLC) equipped with photodiode array and refractive index detectors. Polystyrene (PS) standards and a 1 mL min^{-1} $CHCl_3$ flow rate were used.
